# *Domains of Unknown Function 538-7* Regulates Cotton Resistance to *Verticillium* Wilt by Mediating Jasmonate Signaling Pathways

**DOI:** 10.3390/plants15142148

**Published:** 2026-07-12

**Authors:** Pengtao Li, Yanfang Li, Baomeng Tang, Xiaonan Wang, Siyuan Li, Jiayue Hou, Shuhua Yin, Siyu Lu, Wankui Gong, Yangyang Wei, Quanwei Lu, Yuling Liu, Rui Yang, Yu Chen, Youlu Yuan, Wenkui Wang, Juwu Gong, Renhai Peng

**Affiliations:** 1 School of Biotechnology and Food Engineering, Anyang Institute of Technology, Anyang 455000, China; lipengtao1056@126.com (P.L.); m18737269052@163.com (B.T.); 17838290368@163.com (X.W.); 15670096003@163.com (S.L.); 15536276381@163.com (J.H.); 15516705162@163.com (S.Y.); 16684006509@163.com (S.L.); weiyangyang511@126.com (Y.W.); daweianyang@163.com (Q.L.); liuylay2012@163.com (Y.L.); cyu990324@163.com (Y.C.); 2 The Ministry of Agriculture, Institute of Cotton Research, Chinese Academy of Agricultural Sciences, Anyang 455000, China; gongwankui@caas.cn (W.G.); yuanyoulu@caas.cn (Y.Y.); 3 College of Agriculture, Tarim University, Alar 843300, China; sybks10lyf25@163.com; 4 Xinjiang Production and Construction Crops Seventh Division Agricultural Research Institute, Kuitun 833200, China; 15388465669@163.com

**Keywords:** cotton, *Verticillium* wilt, DUF538-7, QTL interval, VIGS, JA signaling pathway

## Abstract

The *DUF538* gene family, harboring unknown functional proteins, has been reported to take active roles in plant development and response to adversities, while few studies of genome-wide identification and functional verification have been performed in cotton. Hence, two ancestral diploid species, *G. arboretum* and *G. raimondii*, and two cultivated tetraploid ones, *G. hirsutum* and *G. barbadense*, were chosen in this study to investigate the cotton *DUF538* gene family, resulting in 37, 37, 70, and 70 members identified, respectively. A phylogenetic tree was constructed on these cotton *DUF538* genes, together with 22 *A. thaliana* ones, which were divided into seven groups unevenly distributed across nearly all chromosomes. High-degree conservatism, while rich in diversity, was separately observed in gene structure and conserved motif analyses between the same groups and different groups, and a great number of gene-replication events were detected from intraspecific and interspecific collinearity analyses, implying this was the driving force for DUF538 family expansion. Multiple cis-acting elements relevant to adversity-stress responses were found in the promoter region, which were consistent with the transcriptome expression analyses in response to low-temperature and drought stress and *Verticillium* wilt infection. Coincidentally, *GhDUF538-7* showed the core position in the protein–protein interaction network and was identified in the overlapping region of the interval of four reported VW resistance-related QTLs. The gene function of *GhDUF538-7* was verified via gene cloning, relative expression-pattern detection, and virus-induced gene silencing (VIGS) experiment. The *TRV:DUF538-7* plants showed more serious VW symptoms, significantly severe disease indices, relatively higher fungal biomass, and increased brown vascular bundles compared with *TRV:00* plants. Significantly lower expression levels of marker genes *PR4* and *MYC2* in jasmonate signaling pathways indicated *GhDUF538-7* as a potentially positive regulatory factor in plant defense via hormone signal transduction. This study not only broadened the research perspective of evolution and functional differentiation of the cotton *DUF538* gene family, but it also revealed the cooperative relationship between *DUF538-7* and the JA pathway for further molecular mechanisms of cotton resistance to VW infection.

## 1. Introduction

The Domains of Unknown Function (DUFs) proteins refer to proteins whose functional annotations remain unclear, accounting for nearly 23% of the total Pfam families (http://pfam.xfam.org/, accessed on 8 January 2021) [1]. Although the structural and functional analysis of DUFs proteins is a major challenge in the post-genomic era, a large number of *DUF* genes have been reported to make great contributions to the growth and development of plants and their tolerance to adverse conditions [2]. In *Arabidopsis*, all characterized members of the *DUF579* family have an influence on the integrity of xylan that mainly forms the hemicellulose of cell walls in plant. Among this gene family, *AtAGM1* and *AtAGM2* were found to be essential for the 4-O-methylation of glucuronic acid residues in highly glycosylated arabinogalactan proteins (AGPs), thereby modulating AGP structure and function [3,4]. Compared with the wild-type *Arabidopsis* controls, the plants of *DUF761-1* over-expression exhibited pleiotropic developmental defects, such as reduced leaf lamina expansion and shortened primary root length, indicating its regulatory roles in vegetative organ growth and development [5]. A loss-of-function mutation of RUS4, a member of the *DUF647* family, led to a significant reduction in the number of stamens and the expression levels of some transcription factors (TFs) relevant to stamen and pollen maturation in the jasmonic acid (JA) pathway, namely MYB24, MYB57, and MYB108 [6]. Those findings suggested that RUS4 might act upstream in the JA signaling cascade to coordinate reproductive development [7,8]. In rice, ectopic over-expression of *OsDUF946.4* was reported to enhance abiotic stress tolerance by up-regulating key stress-protective genes, notably late embryogenesis abundant (LEA) proteins and the high-affinity potassium transporter HKT1;1, thereby conferring improved resilience to drought and salinity stress [9]. In addition, the ectopic expression of *ZmDUF1645* in rice could enhance yield via the GIF1 pathway, albeit with a compromise in grain quality and cold tolerance, by disrupting starch synthesis (GBSS1/AGPS2) and redox homeostasis [10].

The *DUF538* gene family encodes a group of proteins harboring the conserved unknown domain that typically exhibits a molecular weight of approximately 19–21 kDa with around 170 amino acid residues, which has been widely identified in monocotyledonous and dicotyledonous plants [11,12]. Previous studies have suggested that the *DUF538* genes could participate in multiple growth/development pathways in plants, such as cell proliferation, chlorophyll decomposition, seed germination, and xylem formation, of which the relative *DUF* genes include *At4g0236*, *CaWSCP*, *CU406238*, *Os06g0538900*, *Os01g0210600*, and *Os11g0241700* [13,14,15,16,17]. Furthermore, some DUF538 members were reported to contribute to plant defenses against biotic responses, which were involved in drought, heat, and saline–alkaline stresses in populus, switchgrass, and falx [18,19,20]. To protect plants from biotic infection, *Glyma10g02180*, *Os11g0594800*, and other *DUF538*-domain genes were found to correlate with the resistances of root-knot nematode, *Verticillium* wilt (VW), *Magnaporthe* grisea, *Fusarium head blight*, and *Botrytis* cinerea [21,22,23,24,25].

Cotton (*Gossypium* spp.) is the primary natural fiber source for the global textile industry and plays pivotal roles in sustaining worldwide economic activity [26]. Fiber yield and quality traits, which determine the economic value of cotton, are adversely affected by a wide range of adversities, including drought, soil salinity, chilling, and heat stresses [27,28,29,30]. Beyond those environmental factors, VW, which is mainly caused by the soil-borne fungi *V. dahliae*, represents one of the most devastating vascular diseases threatening global cotton production, consistently ranking among the top three most destructive cotton diseases in China [31,32]. Compounding these challenges, the progressive degradation of arable land quality, escalating water scarcity, and increasing frequency and intensity of extreme climatic events have elevated abiotic and biotic stresses to primary constraints on both cotton yield enhancement and fiber quality improvement.

In this study, four representative *Gossypium* species were collected for comprehensive analyses of *DUF538* genes, and an integrated approach employing comparative genomics, phylogenetic reconstruction, collinearity analysis, and transcriptome mining were conducted for systematic investigation of family membership, evolutionary history, and expression regulatory networks. Meanwhile, for the hub gene *DUF538-7,* further functional verification was performed via quantitative real-time PCR (qRT-PCR) and virus-induced gene silencing (VIGS) experiments, in which the expression pattern and silenced plant phenotype in response to VW infection indicated its potential role in cotton resistance to VW. Therefore, systematically identifying stress tolerance-related genes in cotton and elucidating their functions, while accelerating the translation and application of relevant mechanistic insights into marker-assisted and gene-edited breeding programs, is of critical significance for enhancing the climate resilience of cotton production systems, ensuring the long-term sustainable development of the global cotton sector while safeguarding national food security and the security of strategic agricultural products.

## 2. Materials and Methods

### 2.1. Identification and Sequence–Property Analysis of DUF538 Members

Genome data of *Gossypium* species and *A*. *thaliana* were separately obtained from CottonMD and TAIR database [32,33], of which the complete genomic sequences, coding sequences, protein sequences, and gene annotation files were utilized for bioinformatic analyses. The Hidden Markov Model (HMM) profile IPR008801 of DUF538 family was downloaded from the Pfam database, and candidate sequences were subsequently screened by BLASTP and HMMER 3.0 software [34,35]. The conserved domains in candidate sequences were verified by NCBI Batch CD-Search (http://www.ncbi.nlm.nih.gov/Structure/ccd/wrpsb.cgi, accessed on 1 March 2021) and Search Pfam (http://pfam.xfam.org/, accessed on 6 January 2015) [36,37], finally confirming member information. The locally installed software TBTools v2.0 [38] was utilized to calculate the amino acid length, molecular weight, and theoretical isoelectric point, which were also confirmed by the online website ExPASy (https://web.expasy.org/compute_pi/, accessed on 2 July 2021) [39]. Besides, two online websites, namely as WoLF-PSORT (https://wolfpsort.hgc.jp/, accessed on 1 July 2007) [40] and Cello 2.5 (http://cello.life.nctu.edu.tw/, accessed on 1 April 2022) [41], were chosen in this study to predict the subcellular localization of cotton DUF538 proteins.

### 2.2. Evolutionary-Tree Construction and Chromosomal Locations of DUF538 Family

Multiple sequence alignment was performed between 213 cotton DUF538 proteins and 22 AtDUF538 proteins with the software Clustal X 2.0 [42], and their phylogenetic relationships were interpreted using the neighbor-joining (NJ) method of the software MEGA 7.0, with the Bootstrap value set as 1000 [43]. Subsequently, the online Evolview (https://www.evolgenius.info/evolview/, accessed on 2 July 2019) was utilized to modify the evolutionary tree [44].

Referring to the genomic and annotation information of the 4 cotton species, the chromosome locations of all cotton *DUF538* genes were extracted and subjected to visualization analysis by TBTools v2.0 [38].

### 2.3. Collinearity and Purification-Selection Analyses of Cotton DUF538s

The analyses of interspecific and intraspecific collinearity were performed on the cotton *DUF538* genes by TBTools v2.0 [38], and their collinearity plots were drawn through the software Circos v0.51 [45]. Ka/Ks ratios were calculated for the identification of duplicated gene pairs to infer their prevailing mode of selection. Specifically, Ka/Ks > 1, =1, and <1 indicated positive selection, neutral evolution, and negative (purifying) selection, respectively [38].

### 2.4. Gene Structure and Conserved Motif Analyses of Cotton DUF538 Genes

Based on the extracted location data of cotton DUF538 members, the exon–intron structure of each gene was analyzed using TBtools v2.0, resulting in their structure diagram [38]. The conserved motifs of the DUF538 protein sequence were identified using the online MEME, with the maximum number of motifs, minimum motif length, and maximum length set as 13, 6, and 50, respectively [46]. The visualization drawing of the combined phylogenetic tree, gene structure, and conserved motif of cotton *DUF538* genes was performed by TBTools [38]. The promoter sequences (2000 bp upstream of the initiation codon) were separately extracted from their genomic files, which were subjected to cis-acting element identification by the online database PlantCARE [47].

### 2.5. Expression-Pattern Analyses and Co-Expression Network Construction

To investigate the tissue-specific expression status of cotton *DUF538* genes, the published RNA-seq data of the TM-1 (*G. hirsutum*) and Hai7124 (*G. barbadense*) were separately downloaded from the database Sequence Read Archive (SRA) of the National Center for Biotechnology Information (NCBI). The nine types of tissues were anther, epicalyx, leaf, petal, pistil, root, sepal, stem, and torus, and their uploaded SRA number was PRJNA490626 [48]. Similarly, the development processes of the *UDF538* gene family in cotton ovules and fibers were also monitored in the two above-mentioned genetic-standard varieties, of which samples of the former were collected at 0, 1, 3, 5, and 10 days post anthesis (DPA), and the latter ones were sampled at 10, 20, and 25 DPA [48]. Furthermore, the abiotic response of cotton *UDF538* genes to high-temperature (37 °C), low-temperature (4 °C), drought (PEG6000), and salt (0.4 M NaCl) stresses were explored by the virtue of TM-1 RNA-seq (SRA number: PRJNA248163) [49]. In addition, the expression patterns in response to VW infection were also analyzed in the cotton *DUF538* genes by downloading the transcriptome data of TM-1 and Hai7124 inoculated by *V. dahliae* 991 [50].

A co-expression network of *GhDUF538* genes was constructed for screening the potential hub genes [51], of which each correlation coefficient was calculated by the strength of expression level responding to VW infection [52]. The significant co-expression relationship was defined with the threshold of |r| > 0.7 and *p* < 0.01, while the degree centrality was evaluated by the number of significant correlation connections with other UDF538 members [53]. The interacting network was drawn by linkET of the R package.

### 2.6. Expression Mode and VIGS Verification of DUF538-7 in Response to VW Infection

The VW-resistant CSSL MBI9626 was selected to monitor expression patterns and VIGS verification of DUF538-7 in response to VW infection, of which the seeds were planted in paper cups filled with 6:4 of sand and vermiculite and incubated under 28 °C and 16 h light/8 h dark conditions. When the first true leaf was unfurled, the seedlings were inoculated with 10^7^ spores/mL Vd991 in three biological replicates. The root samples at 0, 1, 2, 3, 5, 7, 10, and 15 days after inoculation (DAI) were separately collected for qRT-PCR experiments. RNA extraction was performed according to the operating manual of FastPure Cell/Tissue Total RNA Isolation Kit V3 (RC122-01, Vazyme, Nanjing, China), which were then subjected to cDNA synthesis by HiScript III RT SuperMix for qPCR (R323-01, Vazyme, Nanjing, China). The ChamQ Universal SYBR qPCR Master Mix (Q711-02, Vazyme, Nanjing, China) was used to perform the qRT-PCR procedure on ABI 7500Fast system (Applied Biosystems, Waltham, MA, USA), and the relative expression levels of the target genes were evaluated using the algorithm 2^−∆∆Ct^ method [54], with three biological and three technological replicates.

The coding sequence of *DUF538-7* was obtained from the online COTTONOMICS (http://cotton.zju.edu.cn/, accessed on 12 September 2022) [55], in which the specific primers were designed by the online SGN-VIGS (http://vigs.solgenomics.net/), accessed on 1 August 2009. The tobacco rattle virus2 (TRV2) vector was used to construct the *TRV:GhDUF538-7* plasmid, which was subsequently transformed into agrobacterium tumefaciens stain LBA4404 (G6038, AngYuBio, Shanghai, China) [52]. The VW-resistant CSSL MBI9626 and VW-susceptible *G. hirsutum* CCRI36 were utilized for agrobacterium injection, which was performed when the cotton seedlings grew to the stage when two cotyledons were fully expanded. Two weeks after the treatment, true leaves were sampled to conduct qRT-PCR experimentation for calculating silencing efficiency, while the seedlings were placed in a pulp tray with 2 mL spore suspension (10^7^ spores/mL Vd991) for inoculation. Disease phenotypes were counted at 21 DAI, and the disease index (DI) was calculated using the formula [∑(Ni * i)/(N * 4)] * 100, where Ni, i, and N represent the number of seedlings at the corresponding disease grade, the disease grade, and total seedling number, respectively [52]. The VW severity is classified into five grades:

Grade 0: healthy plants with no wilting or defoliation symptoms leaves.

Grade 1: 1–2 cotyledons are wilted, while no symptoms are observed on true leaves.

Grade 2: 2 cotyledons are wilted, with 1 true leaf showing disease symptoms.

Grade 3: 2 true leaves exhibit disease symptoms.

Grade 4: 3 or more true leaves show disease symptoms; in severe cases, all leaves of the plant defoliate and the apical meristem dies [52].

In addition, 2 cm long stem segments are excised from the diseased plants after 30 DAI. The segments were divided into two aliquots, one of which was subjected to longitudinally sectioning to observe the vascular bundle status under a stereomicroscope, while the other was placed on the potato dextrose agar (PDA) solid medium for fungal recovery culture [56]. The fungal biomass quantification was conducted by randomly sampling the 2nd infected true leaf, which was used for fungal DNA template to perform qRT-PCR experimentation with *V*. *dahliae*-specific primers ITS1-F and ST-Ve1-R [57].

Given that a large number of JA-related cis-acting elements were identified in the promoter region, *pathogenesis-related protein 4* (*PR4*) [58] and *MYC2* [59] were selected as the marker genes in JA signaling pathways to further investigate the molecular mechanisms of cotton resistance to VW infection. The specific primers of *GhPR4* and *GhMYC2* were designed using SGN-VIGS (http://vigs.solgenomics.net/, accessed on 1 August 20), and qRT-PCR experiments were performed on *TRV:DUF538-7* and *TRV:00* MBI9626 plants to assess their relative expression levels.

## 3. Results

### 3.1. Genome-Wide Identification and Analyses of Cotton DUF538 Genes

**Figure 1:**
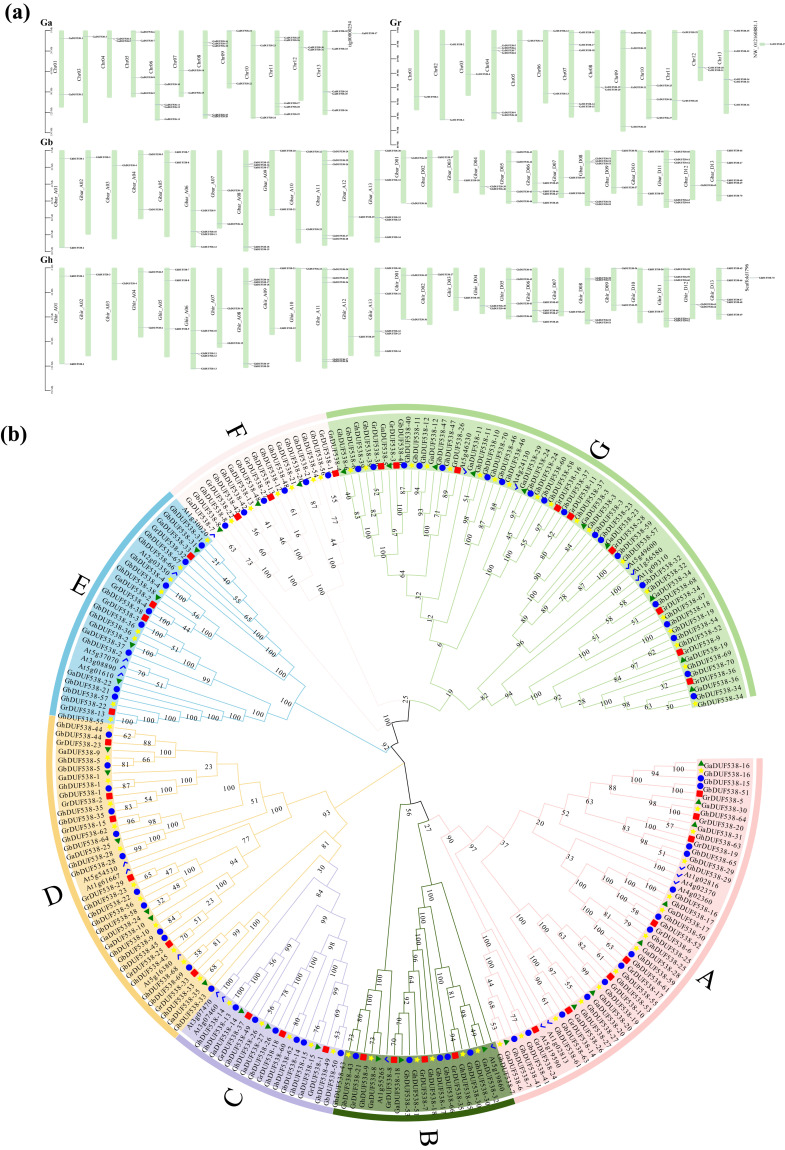
The chromosomal location and phylogenetic tree of the *DUF538* gene family. (**a**) The chromosomal location of all cotton *DUF538* genes of the four representative *Gossypium* species. (**b**) The maximum likelihood phylogenetic tree of DUF538 proteins from *A. thaliana* and *Gossypium* species. The different symbols represented the diverse groups of DUF538 genes, among which the blue cuntermark represented the AtDUF538 genes, the green triangle represented the GaDUF538 genes, the red quadrangle represented the GrDUF538 genes, the yellow star represented the GbDUF538 genes, and the blue circle represented the GbDUF538 genes.

A total of 214 *DUF538* genes were identified across four representative *Gossypium* species, including 70 *G. hirsutum*, 70 *G. barbadense*, 37 *G. arboreum*, and 37 *G. raimondii*, which were sorted in terms of their chromosomal locations and named *GhDUF538-1* to *GhDUF538-70*, *GbDUF538-1* to *GbDUF538-70*, *GaDUF538-1* to *GaDUF538-37*, and *GrDUF538-1* to *GrDUF538-37*, respectively (Figure 1a). In diploid genomes, *DUF538* genes were distributed across almost all 13 chromosomes, except for the second chromosome in *G. arboreum*. Among these, the eighth, 11th, and 13th chromosomes of *G*. *arboretum* harbored the maximum number of *GaDUF538* genes, while the fourth, seventh, and 13th chromosomes of *G*. *raimondii* harbored the maximum number of *GrDUF538* genes. In the tetraploid genomes, the *DUF538* genes were distributed across all 26 chromosomes, but their distribution was uneven. The common feature between *G. hirsutum* and *G. barbadense* was that their chromosomes A08, A11, A13, D11, and D13 harbored the maximum number of *DUF538* genes (five genes on each chromosome); the difference between them lied on chromosome D08, in which *G*. *hirsutum* harbored five *GhDUF538* genes.

The protein lengths of all identified cotton DUF538s ranged from 89 (GhDUF538-70) to 244 (GaDUF538-2), and their molecular weights (MWs) ranged from 10.28 (GhDUF538-70) to 27.25 (GaDUF538-2). The minimum (4.2) isoelectric point (Ip) value was observed between GrDUF538-6 and GhDUF538-50, while GhDUF538-38 showed the maximum Ip value (9.8). Approximately 68% of cotton DUF538 members (144 of all 214) presented less than 40 instability indices, indicating their relative stability. The range of aliphatic indices of cotton DUF538 proteins was from 51.45 (GhDUF538-24) to 117.56 (GhDUF538-5), nearly half of which harbored negative values of grand average of hydropathicity (GRAVY). The subcellular localization prediction results of all cotton *DUF538* genes showed that they are distributed in multiple different locations, including 64 in chloroplasts, 56 in cytoplasm, 54 in extracellular spaces, 29 in nuclei, six in plasma membranes, and five in vacuoles (Table S1).

The evolutionary relationships investigated between 214 cotton DUF538s and 22 *A. thaliana* revealed that they were divided into seven subgroups (Figure 1b). Among these, Groups A and G contained the largest number of cotton DUF538s (44 and 59) and AtDUF538s (five and five). The minimum number of DUF538s was observed in Groups B and F, the former of which contained 18 cotton and two *Arabidopsis* DUF538 proteins, while the latter contained 18 cotton and one *Arabidopsis* DUF538s.

### 3.2. Structural-Diversity and Functional-Conservation Analyses of Cotton DUF538 Genes

**Figure 2:**
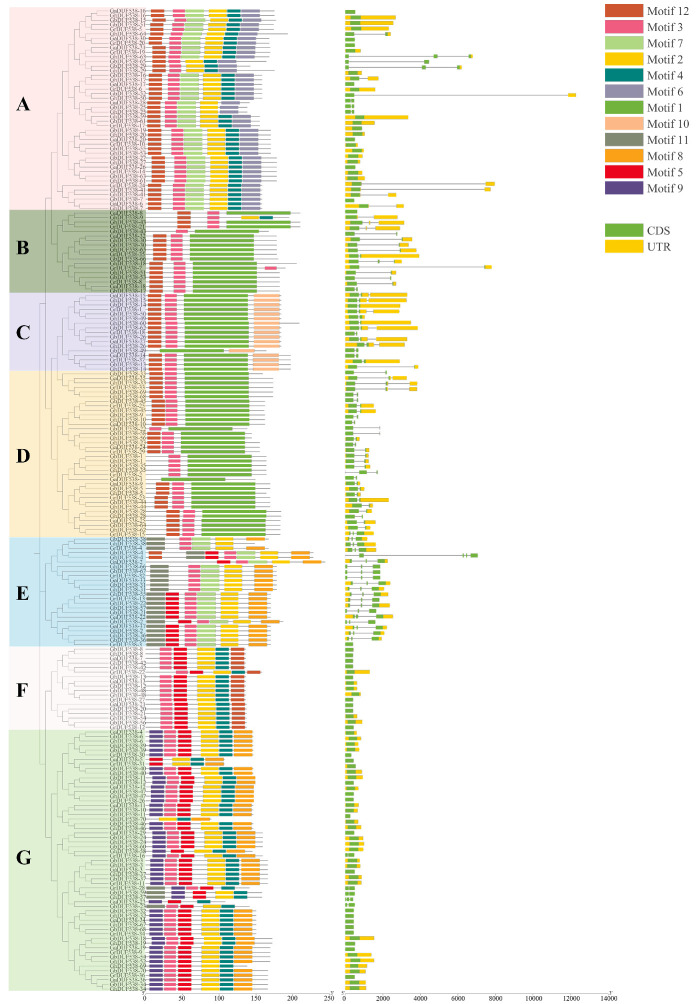
The analyses of evolutionary relationships, gene structures, and conserved motifs of the cotton *DUF538* gene family. (**a**) The evolutionary relationships of the 214 cotton *DUF538* genes. (**b**) The exon–intron structures of 214 cotton *DUF538* genes. (**c**,**d**) The 12 conserved motifs of 214 cotton *DUF538* genes.

To further comprehend their evolutionary relationships, a phylogenic tree was constructed with all the cotton *DUF538* genes (Figure 2a). The results exhibited the same clustering as those combined with *AtDUF538* genes (Figure 1b). Meanwhile, the conserved motif analysis revealed that 12 kinds of conserved motifs, named motif 1 through to motif 12, were identified across the different subgroups (Figure 2d). It was observed that the number of conserved motifs ranged from one (GaDUF538-1) to seven (GhDUF538-4 and GbDUF538-4) in each cotton DUF538 protein, and the same subgroup exhibited a similar conserved motif composition and arrangement, such as motifs 2–5 and motif 12 in Group F, suggesting that members of the *DUF538* gene family clustered in the same subgroup might have similar biological functions. There were no identical conserved motifs contained in all cotton DUF538s, even though 96.26% of these (206 of 214) contained the conserved motif 12. Some specific motifs were only contained in particular subgroups, such as motif 6 in Group A, motif 10 in Group C, and motif 9 in Group G. Similar conserved motif composition of each group of cotton DUF538 proteins were observed in their phylogenetic analysis, indicating the reliable classification of the DUF538 gene family.

Furthermore, the exon–intron structure analysis of all cotton *DUF538* genes (Figure 2c) indicated that the range of intron numbers was from zero to four, and most of cotton DUF538 genes (80 of 214 ones) contained no introns. In addition, 12.62% of the cotton *DUF538* genes (27 of 214 ones) comprised only one exon without UTR. Collectively, the similar exon–intron distribution patterns and intron numbers were observed in the same subgroup of cotton DUF538 genes, while some structural diversity between the different subgroups implied their functional differentiations.

### 3.3. Intraspecific and Interspecific Collinearity Analyses of Cotton DUF538 Genes

**Figure 3:**
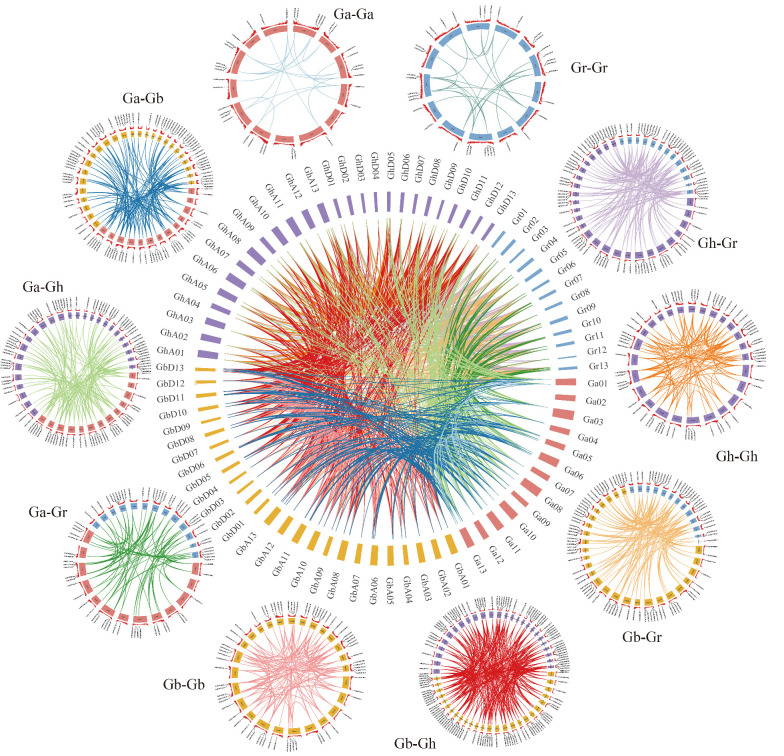
Collinearity analysis of intraspecific and interspecific species of cotton *DUF538* genes.

Gene-replication events are regarded as a principal element for gene family expansion; therefore, the collinearity analyses of *DUF538* genes were conducted on four representative *Gossypium* species (Figure 3). The results demonstrated that tandem repeat events of cotton *DUF538* genes were ubiquitous in intraspecific and between interspecific alignments, indicating their great significance for the evolution and expansion process of this gene family. This result was further supported by the results of intraspecific collinearity analysis, and there were 74, 20, 105, and 106 repeated gene pairs from the groups of *GaDUF538*-*GaDUF538*, *GrDUF538*-*GrDUF538*, *GhDUF538*-*GhDUF538*, and *GbDUF538*-*GbDUF538*, respectively. These collinear relationships indicated that cotton *DUF538* genes might have undergone sequence duplication in chromosomes and maintained a relatively high degree of conservation during evolution. The expansion mechanism of cotton *DUF538* genes might be involved in multiple biological processes, including gene duplication, segmental duplication, transposition, retrotransposition, and chromosomal crossover events, which have jointly shaped the current distribution and composition characteristics of this gene family in the cotton genome.

To further explore the evolutionary origin of *DUF538* genes in cotton, a genome-wide interspecific collinearity analysis was performed on four representative *Gossypium* species. The most repeated gene pairs (254) were identified in *GhDUF538*-*GbDUF538*, while the least (75) were observed in the *GrDUF538*-*GaDUF538* group. Almost the same number of repeat gene pairs were observed in the collinearity analyses between diploid and tetraploid species: there were 122 repeat gene pairs in group *GaDUF538*-*GhDUF538*, 126 pairs in group *GaDUF538*-*GhDUF538*, and 155 pairs in both *GrDUF538*-*GhDUF538* and *GrDUF538*-*GbDUF538*. These conserved collinearity relationships observed between diploid and tetraploid indicated that members of cotton *DUF538* gene family might be retained during the subsequent polyploidization and genomic evolution of *Gossipium* genus, which laid the foundation for the expansion and functional diversification of this gene family.

### 3.4. Cis-Acting Element Analyses of Cotton DUF538 Genes

**Figure 4:**
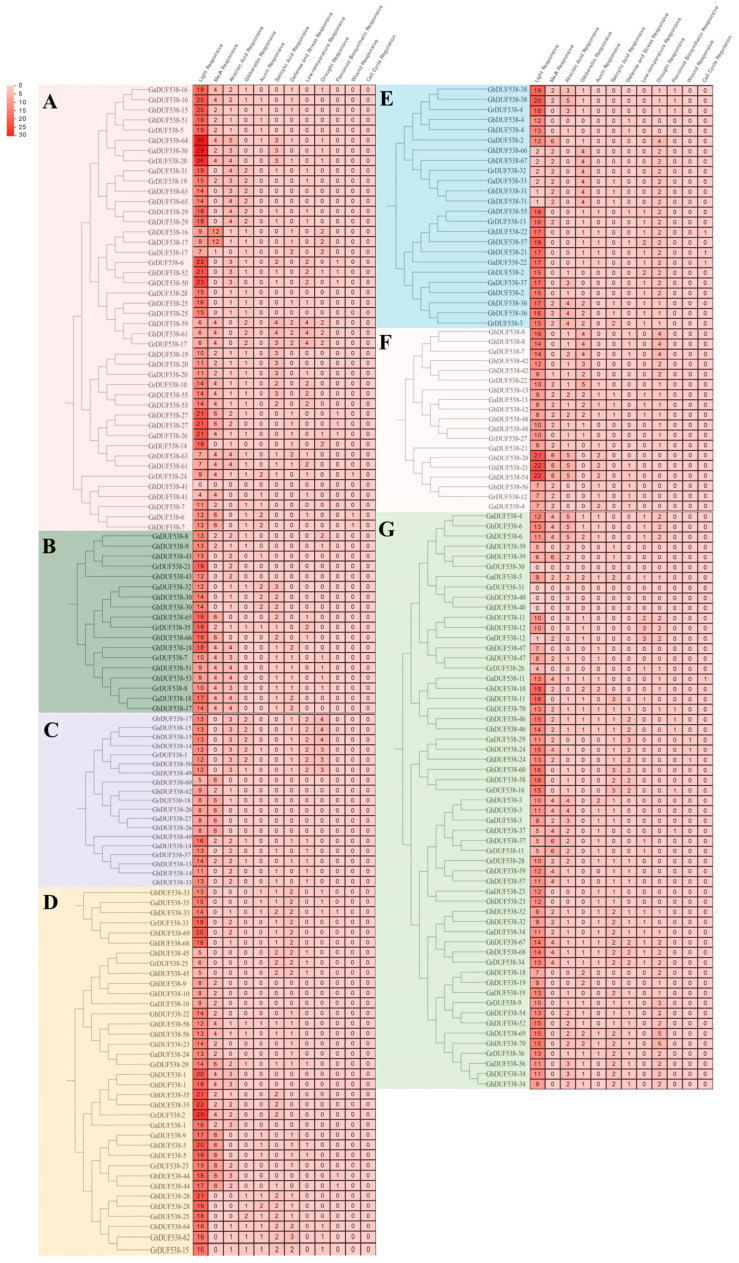
The identification of cis-acting elements in the promoter regions of cotton *DUF538* genes. All the cotton Duf538 genes were divided into seven groups, namely as Gropu A to Group G.

To deeply dissect the potential functions of cotton *DUF538* genes, the cis-acting elements that could play a crucial role in gene expression regulation within the 2000 bp promoter regions, upstream of their transcription start sites (TSSs), were predicted. The results indicated the enriched cis-acting elements in their promoter regions were divided into three major categories, namely phytohormone responsive, plant growth and development, and biotic/abiotic stress responsive; among these, the top 12 elements from the highest to lowest were as follows: light responsive, MeJA responsive, abscisic acid responsive, gilbberellim responsive, auxin responsive, salicylic acid responsive, defense and stress responsive, low-temperature responsive, drought responsive, flavonoid biosynthetic responsive, wound responsive, and cell cycle regulation (Figure 4). Collectively, the cis-acting element of light responsive was found to be present in nearly all cotton *DUF538* genes except for *GbDUF538-41* (Group A), as well as *GrDUF538-31*, *GrDUF538-30*, *GbDUF538-40*, and *GhDUF538-40* (Group G), accounting for the largest numbers of the total cis-acting elements. In addition, the *DUF538* genes of Groups B, C, and G did not contain the cis-acting elements of flavonoid biosynthetic responsive, wound responsive, and cell cycle regulation, implying their roles might have little relation with plant growth and development.

### 3.5. Analyses of Tissue-Specific Expression and Patterns to Abiotic/Biotic Stresses

**Figure 5:**
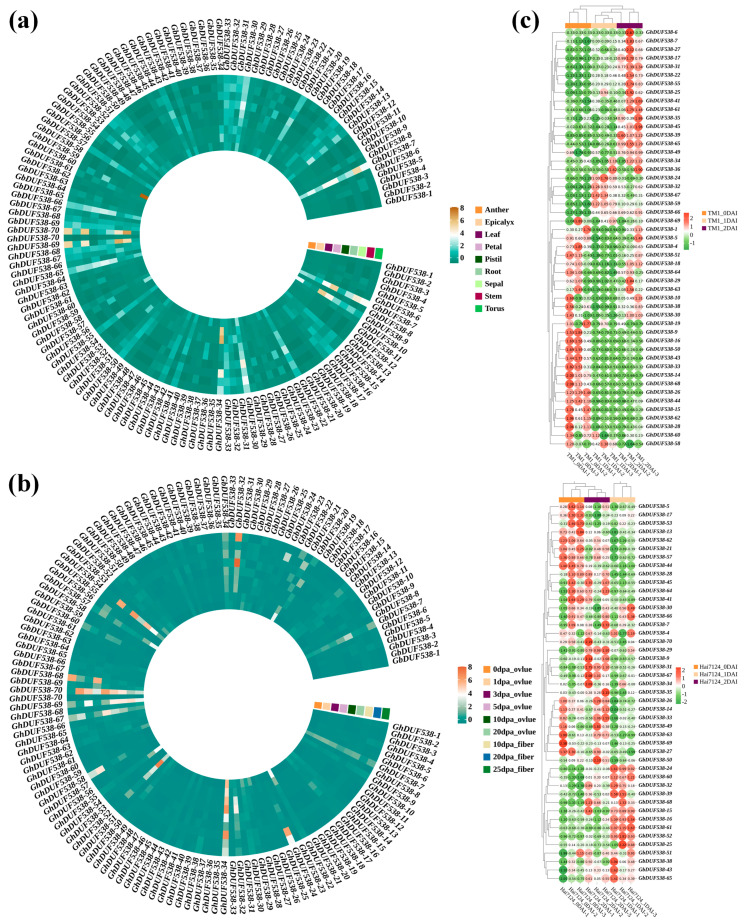
The analyses of tissue-specific expression and responding patterns to VW infection of *GhDUF538* and *GbDUF538* genes. (**a**) The heatmap of tissue-specific expression of *GhDUF538* genes. (**b**) The heatmap of tissue-specific expression of *GbDUF538* genes. (**c**) The heatmap of responding expression of *GhDUF538* and *GbDUF538* genes to VW infection.

The public transcriptome data of cotton anther, epicalyx, leaf, petal, pistil, root, sepal, stem, and torus were utilized for exploring the tissue-specific expression of cotton *DUF538* genes [48]. The results indicated that *GhDUF538-7*, *GhDUF538-9*, *GhDUF538-22*, *GhDUF538-30*, *GhDUF538-34*, *GhDUF538-65*, and *GhDUF538-69* showed higher expression levels in all the nine TM-1 tissues, while *GhDUF538-47*, *GhDUF538-54*, *GhDUF538-56*, and *GhDUF538-70* did not express these at all (Figure 5a). To be specific, *GhDUF538-34*, *GhDUF538-69* and *GhDUF538-7* showed the maximum FPKM values in epicalyx, leaf, sepal, and torus, while *GhDUF538-9* and *GhDUF538-7* were the most highly expressed in anther, petal, and pistil. In TM-1 root, the top two expression genes were *GhDUF538-30* and *GhDUF538-65*, while in TM-1 stem, the top two expression genes were *GhDUF538-7* and *GhDUF538-30*. In Hai724, *GbDUF538-7*, *GbDUF538-22*, *GbDUF538-30*, *GbDUF538-65*, *GbDUF538-68*, and *GbDUF538-39* had higher expression levels in the nine tissues (Figure 5b), among which *GbDUF538-7* and *GbDUF538-39* showed the maximum FPKM values in epicalyx, leaf, petal, sepal, and torus. In addition, *GbUDF538-7* exhibited the highest expression level in pistil, sepal, and stem of Hai7124, while in anther, this gene showed a comparable expression level to *GbDUF538-58*. Overall, either *GhDUF538-7* or *GbDUF538-7* was stable and highly expressed in all nine tissues of TM-1 and Hai7124, implying their potential roles participating in plant growth and development.

The responding patterns of cotton *DUF538* genes to biotic stress were also investigated via RNA-seq technology using TM-1 and Hai7124 after inoculation with *V. dahliae* 991 [50], and the results showed diverse expression changes in *DUF538* genes in response to VW infection (Figure 5c). In TM-1, a total of 12 cotton *DUF538* genes were highly expressed from 0 to 2 DAI, namely *GhUDF538-7*, *GhUDF538-9*, *GhUDF538-14*, *GhUDF538-16*, *GhUDF538-22*, *GhUDF538-30*, *GhUDF538-33*, *GhUDF538-43*, *GhUDF538-50*, *GhUDF538-55*, *GhUDF538-65*, and *GhUDF538-68*. Among these highly expressed genes, *GhUDF538-7*, *GhUDF538-22*, *GhUDF538-55*, and *GhUDF538-65* showed a continuously up-regulated expression trend to cope with VW infection, while the other eight *GhDUF538* genes presented a constantly down-regulated pattern in response to Vd991 inoculation. The highly expressed DUF538 genes in Hai7124 included *GbDUF538-7*, *GbDUF538-14*, *GbDUF538-17*, *GbDUF538-21*, *GbDUF538-24*, *GbDUF538-30*, *GbDUF538-33*, *GbDUF538-43*, *GbDUF538-53*, *GbDUF538-57*, *GbDUF538-60*, and *GbDUF538-66*. Among these, *GbDUF538-7*, *GbDUF538-9*, *GbDUF538-30*, and *GbDUF538-66* showed a constant up-regulation from 0 to 2 DAI, while *GbDUF538-17*, *GbDUF538-21*, *GbDUF538-53*, and *GbDUF538-57* showed a continuous down-regulation. In addition, *GbDUF538-13* and *GbDUF538-33* exhibited an expression pattern that was first down-regulated and then up-regulated, while *GbDUF538-43* exhibited the opposite expression trend (first up-regulated and then down-regulated). These cotton *DUF538* genes, possessing the potential functions of stable positive or negative regulation on VW resistance, might require more attention.

**Figure 6:**
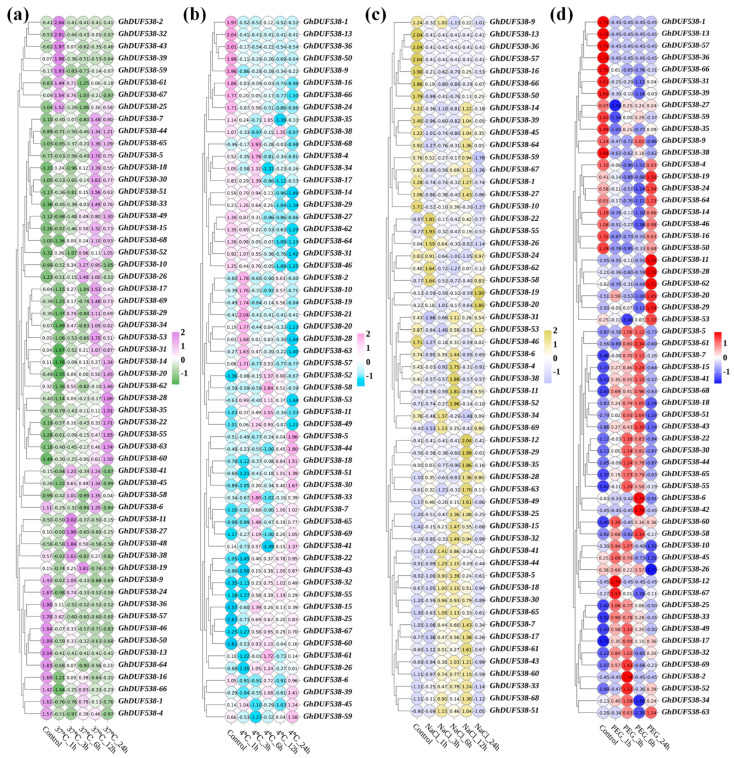
The analyses of responding patterns of *GhDUF538* genes to multiple abiotic stresses. (**a**) The heatmap of responding expressions of *GhDUF538* genes to high-temperature stress. (**b**) The heatmap of responding expressions of *GhDUF538* genes to low-temperature stress. (**c**) The heatmap of responding expressions of *GhDUF538* genes to salt stress. (**d**) The heatmap of responding expressions of *GhDUF538* genes to drought stress.

To make the *G. hirsutum* varieties exhibit greater resilience to adverse stresses, TM-1 RNA-seq data in response to multiple abiotic stresses at 0, 1, 3, 6, 12, and 24 h were utilized to screen candidate genes of the cotton *DUF538* family [49]. The results showed that *GhDUF538-7*, *GhDUF538-15*, *GhDUF538-17*, *GhDUF538-22*, *GhDUF538-30*, *GhDUF538-31*, *GhDUF538-34*, *GhDUF538-51*, *GhDUF538-55*, *GhDUF538-65*, and *GhDUF538-69* exhibited higher expression levels in response to high-temperature environments, while *GhDUF538-3*, *GhDUF538-8*, *GhDUF538-12*, *GhDUF538-21*, *GhDUF538-23*, *GhDUF538-37*, *GhDUF538-42*, *GhDUF538-47*, *GhDUF538-54*, *GhDUF538-56*, and *GhDUF538-70* were not expressed during the 37 °C treatment process (Figure 6a). Among these highly expressed genes, it was observed that *GhDUF538-7*, *GhDUF538-15*, *GhDUF538-30*, *GhDUF538-51*, and *GhDUF538-65* showed up-regulation first and then down-regulation as an expression trend, with the peak FPKM value at 12 h; meanwhile, *GhDUF538-17*, *GhDUF538-31*, *GhDUF538-34*, and *GhDUF538-69* exhibited a more complicated expression pattern with the same maximum FPKM at 12 h, in which the genes first exhibited down-regulation, followed by up-regulation, and then a final down-regulation. In addition, *GhDUF538-22* and *GhDUF538-55* were continuously up-regulated in response to heat stress, reaching the peak expression level at 24 h. In total, 12 cotton *DUF538* genes were screened with higher expression levels during the low-temperature stress (4 °C), namely *GhDUF538-7*, *GhDUF538-15*, *GhDUF538-17*, *GhDUF538-22*, *GhDUF538-30*, *GhDUF538-34*, *GhDUF538-41*, *GhDUF538-43*, *GhDUF538-51*, *GhDUF538-55*, *GhDUF538-65*, and *GhDUF538-69*, among which *GhDUF538-7*, *GhDUF538-30*, *GhDUF538-41*, *GhDUF538-65*, and *GhDUF538-69* showed expression patterns of up-regulation first, then down-regulation, and finally up-regulation; meanwhile, *GhDUF538-17* and *GhDUF538-34* showed the opposite expression trends (first down-regulation, then up-regulation, and finally down-regulation) (Figure 6b). Furthermore, *GhDUF538-15*, *GhDUF538-22*, *GhDUF538-43*, and *GhDUF538-55* first exhibited first up-regulation and then down-regulation expression trends, and *GhDUF538-31* showed an expression pattern of down-regulation first and then up-regulation in response to 4 °C stress. Under salt stress, it was observed that *GhDUF538-7*, *GhDUF538-17*, *GhDUF538-22*, *GhDUF538-30*, *GhDUF538-31*, *GhDUF538-34*, *GhDUF538-41*, *GhDUF538-55*, *GhDUF538-65*, and *GhDUF538-69* were highly expressed from 0 to 24 h, among which *GhDUF538-22*, *GhDUF538-30*, *GhDUF538-41*, *GhDUF538-55*, and *GhDUF538-65* showed an expression pattern of up-regulation first and then down-regulation, while *GhDUF538-7* and *GhDUF538-17* showed down-regulation first, then up-regulation, and finally down-regulation (Figure 6c). The most complex expression trend was observed in *GhDUF538-31*, *GhDUF538-34*, and *GhDUF538-69*, which were down-regulated first, up-regulated second, down-regulated third, and then finally up-regulated. Under drought stress, the highly expressed genes included *GhDUF538-7*, *GhDUF538-17*, *GhDUF538-22*, *GhDUF538-30*, *GhDUF538-31*, *GhDUF538-34*, *GhDUF538-41*, *GhDUF538-55*, *GhDUF538-65*, and *GhDUF538-69*. Most of these first showed up-regulation and then down-regulation expression patterns in response to PEG6000 treatment—only *GhDUF538-31* was first down-regulated and then up-regulated (Figure 6d). In conclusion, cotton *DUF538* genes might take significant roles in plant adaption to the complex and ever-changing external environments.

### 3.6. Identification and Functional Verification of a Hub GhDUF538-7 Gene in Cotton

**Figure 7:**
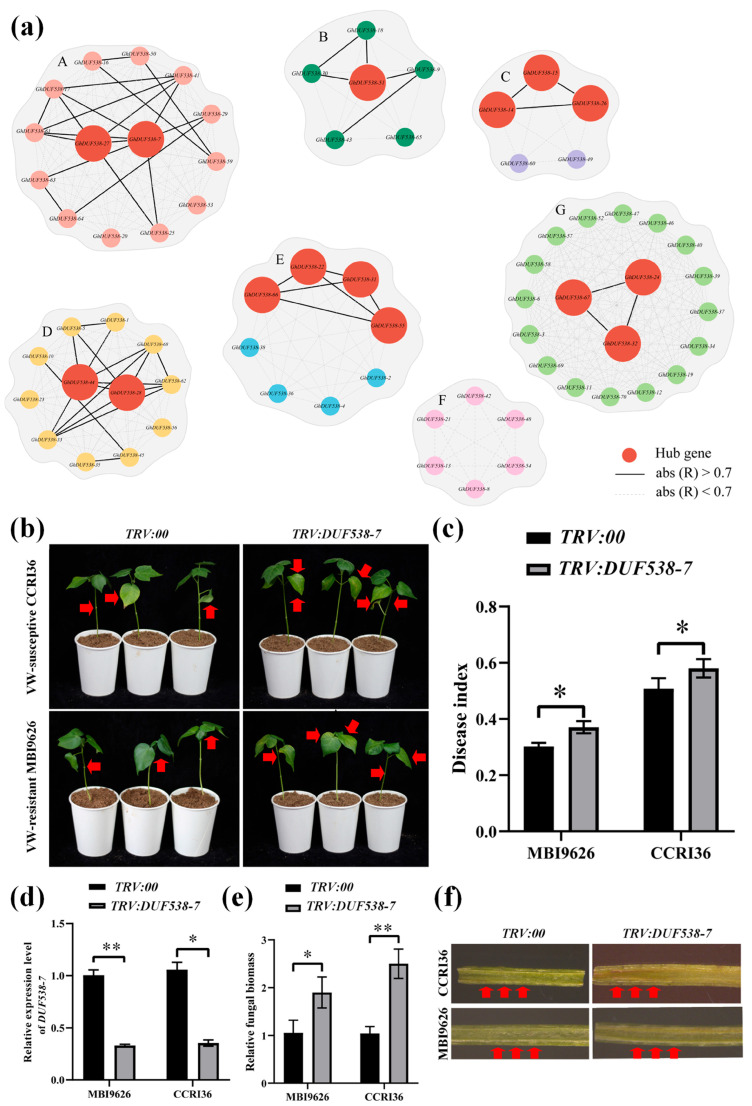
The construction of a protein–protein interaction network of *GhDUF538* genes and functional verification of *GhDUF538-7* in response to VW infection. (**a**) The PPI network of GhDUF538 proteins. (**b**) The VW phenotypes of TRV:DUF538-7 and TRV:00 in MBI9626 and CCRI36. (**c**) The disease indices of TRV:DUF538-7 and TRV:00 in MBI9626 and CCRI36. (**d**) The relative expression levels of candidate genes of TRV:DUF538-7 and TRV:00 in MBI9626 and CCRI36. (**e**) The relative fungal biomass of TRV:DUF538-7 and TRV:00 in MBI9626 and CCRI36. (**f**) The sections of vascular bundle of TRV:DUF538-7 and TRV:00 in MBI9626 and CCRI36. * and ** represented the significant (*p* < 0.01) and extremely significant (*p* < 0.01) differences, respectively, and the red arrows represented the VW symptoms in the leaves and stems.

Aiming at deeper exploration of the potential candidate genes that play significant roles in regulating plant development and resistance against adverse stresses, co-repression networks were performed for all 70 *GhDUF538* genes for hub gene identification (Figure 7a). In Group A, GhDUF538-7 and GhDUF538-27 occupied the core position in the protein–protein interaction network, and similar core proteins also included GhDUF538-31 in Group B, GhDUF538-28 and GhDUF538-44 in Group D, and GhDUF538-24, GhDUF538-32, and GhDUF538-67 in Group G. Despite not being in the core position, *GhDUF538-14*, *GhDUF538-15*, and *GhDUF538-26* in Group C and *GhDUF538-22*, *GhDUF538-33*, *GhDUF538-55*, and *GhDUF538-66* in Group E were screened as hub genes in the co-expression network. However, there were no hub genes in Group F.

Based on the above-mentioned analysis, *GhDUF538-7*, *GhDUF538-22*, and *GhDUF538-69* were identified to be commonly and highly expressed either in the nine tissues or in response to abiotic and biotic stresses. In combination with the results of the co-expression networks, *GhDUF538-7* was regarded as a crucial candidate gene for further functional verification. Referring to the previous QTL studies, *GhDUF538-7* (GH_A05G0221) was located in the overlapping interval of *qVW-C5-2* [60], *qVW-Chr05-1* [61], *qVW-A5-1* [62], and *qRDI-A05-1* [63]. Its promoter region contained multiple phytohormone-responsive elements, including MeJA, gibberellin, and auxin, which have been proven to positively participate in plant defense against pathogenic fungal infection [64,65,66], further supporting the potential role of this gene in cotton VW resistance. Therefore, the association between GhDUF538 and VW resistance was investigated via a series of molecular biology experiments using a stable VW-resistant line MBI9626 and its parents, the VW-resistant G. barbadense variety Hai1 and the VW-susceptible G. hissutum variety CCRI36 [52]. After aligning the three *DUF538-7* coding sequences (CDs, Figure S1c), which were separately cloned from three materials via RT-PCR, with the assembled references (TM-1 and Hai7124), it was observed that the sequences of MBI9626, CCRI36, and TM-1 were completely identical, and the sequences of Hai1 and Hai7124 were identical (Figure S1a). Two SNPs were identified at 277 bp and 340 bp positions between the sequences of TM-1 and Hai7124, of which the latter caused non-synonymous mutation between the two lines (Figure S1b). These findings implied that the *DUF538-7* in MBI9626 might come from *G. hirsutum* lines and not an introgressed gene from *G. barbadense* Hai1. Meanwhile, the expression patterns of *DUF538-7*, in response to Vd991 inoculation from 0 to 15 DAI in MBI9626 via qRT-PCR, showed an expression trend that was first up-regulated (from 0 to 1 DAI), followed by down-regulation (from 1 to 2 DAI), then up-regulation (from 2 to 3 DAI), and finally down-regulation (from 3 to 15 DIA). It was also observed that the expression levels of this gene reached the minimum and maximum values at 0 and 3 DAI, respectively, indicating that this candidate gene might play a more active role in early defense processes of plants against pathogen infection.

VIGS experimentation of *GhDUF538-7* revealed that, two weeks after injection, the cotton seedlings of the positive control showed an albinism phenotype in the leaves (Figure S1c), indicating that the VIGS system could function normally within cotton plants. Both CSSL MBI9626 and CCRI36 were selected to perform VIGS experiments (Figure 7d). After 21 DAI of Vd992 inoculation at the concentration of 10^7^ spores/mL, the *TRV:DUF538-7* plants showed more severe VW symptoms along with more true leaves turning yellow and falling down compared to the *TRV:00* plants of CCRI36 and MBI9626. The result was consistent with the DI investigation results, in which the DIs of *TRV:DUF538-7* plants of CCRI36 (57.04) and MBI9626 (37.06) were significantly higher than those of TRV:00 plants of CCRI36 (50.78) and MBI9626 (33.06), respectively (Figure 7c). In addition, it was observed that the relative pathogen biomasses of *TRV:00* leaves of CCRI36 and MBI9626 were significantly lower than those of *TRV:DUF538-7* leaves in the two lines (Figure 7e). Meanwhile, observation of stem cross-sections showed that the vascular bundles in the stems of *DUF538-7*-silenced plants exhibited more significant browning than those of the *TRV:00* control plants (Figure 7f). Collectively, those results indicated that *DUF538-7* might play great roles in plant defense against VW infection. In addition, the relative expression levels of JA-related PR4 and MYC2 were also detected in significantly lower expressions in *TRV:DUF538-7* plants than in *TVR:00* plants (Figure S2), implying the crucial roles of *DUF538-7* in cotton resistance to VW infection via mediating JA signaling pathway.

## 4. Discussion

In recent years, chromosome-level studies and the reference genome-level annotation database of multiple model plants, including *A. thaliana* [67], *Oryza sativa* [68], *Setaria italica* [69], *Brachypodium distachyon* [70], and *Zea mays* [71], have been constantly improved and updated, along with the continuous upgrading and iteration of genome sequencing technologies. Some non-model plants also attracted researchers’ attentions due to their unique traits and biological values, such as *Gossypium* species known to be renewable sources of natural fibers and salt-tolerant pioneers [72], of which their representative genome assemblies were subjected to Next-Generation Sequencing (NGS) [73,74,75], Third-Generation Sequencing (TGS) [76,77,78,79], and Oxford nanopore Technologies (ONTs) sequencing [80,81,82]. Meanwhile, the proportion of *DUF* gene families in the Pfam database has also increased from version 32.0 to 37.0 [83,84], and accumulating studies have shown that the *DUF* gene family is involved in various plant life activities, particularly growth, development, and responses to diverse adverse stress factors [84,85,86]. By utilizing bioinformatic methods to identify the family members, to analyze the physicochemical properties, and to predict the cis-acting elements, in combination with tissue-specific expression analyses and responses to adversity stresses, it is of significant relevance to acknowledge the origins, diversity, and biological functions of the *DUF* gene family [87,88,89,90]. Among the numerous *DUF* gene families in the Pfam database, *DUF538* genes encode a type of highly conservative and plant-specific proteins with potential functions relevant to chlorophyll hydrolysis and pectin methylesterase activity [91,92], which have been reported to respond to multiple adversity stresses and participate in plenty of physiological and biochemical pathways [93,94].

Given its potential functions in plant development and growth, as well as biotic/abiotic stress response, the cotton *DUF538* gene family was selected in the current study to conduct the genome-wide member identification, of which the comprehensive analyses were performed on their chromosomal localization, gene structures, physicochemical properties of the encoding protein, and cis-acting elements in the promoter region in combination with bioinformatic methods. In two ancestral species, 37 *G. arboreum* (*GaUDF538-1* to *GaDUF538-37*) and 37 *G. raimondii* (*GrDUF538-1* to *GrDUF538-37*), members were identified and located in the same 12 chromosomes (none in the second chromosome), of which the *GaUDF538* members encoded 107–244 amino acids (AAs), showed 12.46–27.25 MWs, and harbored 4.43–9.5 Ip values, while the *GrUDF538* members encoded 107–210 AAs, 12.5–23.65 MWs, and 4.2–9.57 Ip values (Table S1). Most of the diploid DUF proteins were predicted with high stability and subcellular localization in extracellular spaces, cytoplasm, and chloroplasts, while half of these belonged to hydrophobic proteins. Compared with the previous study, which only identified 17 *DUF538* genes in *G. raimondii* [86], the current study obtained twenty additional ones that might be attributed to the selection of a reference genome, with more complete chromosome assembly and more comprehensive gene annotation [32]. Nearly twice the DUF538 members were identified in the two representative tetraploid species (70 each), which were found to be unevenly distributed in all 26 chromosomes, among which A08, A11, A13, D11, and D13 chromosomes harbored the largest numbers of *GhDUF538* and *GbDUF538* genes (Figure 1a). The majority of proteins encoded by tetraploid *DUF538* are stable due to these proteins, have less than 40 instability indices (Ins), and more than 80 aliphatic indices (AIs), half of which were hydrophobic proteins. In addition, multiple GhDUF538 and GbDUF538 proteins were located in nuclei except for extracellular spaces, cytoplasm, and chloroplasts. These findings were indicative of the conservative characteristics of DUF538 members during the process of evolution from diploid to tetraploid, while rich diversities might evolve more roles in the development and growth of plants.

In the phylogenetic analysis, family members in the same subgroups showed a similar conserved motif composition and exon–intron structure, such as motifs 2–5 and motif 12 in Group F (Figure 2b), or all 18 *DUF538* genes in Group F without introns (Figure 2c). These results indicated that, during the evolutionary process of the DUF538 gene family, functional diversity might have been achieved while maintaining the conservation of the core domain through variations in the number of exons/introns, reshaping the UTRs and modular recombination of motifs. This structure feature of “core conserved–peripheral variable” is similar to the evolutionary strategy of the *DUF538* family in 13 terrestrial plants [86], and it is also a universal paradigm for the functional differentiation of plant gene families. In addition, the intraspecific collinearity analysis of *DUF538* genes among the four representative cotton species, comparing the diploid–diploid with tetraploid–tetraploid alignment, identified more repeated gene pairs; for example, 105 GhUDF538-GhDUF538 and 106 GbDUF538-GbDUF538 gene pairs were identified (Figure 3). The interspecific collinearities of cotton *DUF538* genes showed that the largest and fewest pairs of repeated genes were observed in the alignment groups of GhDUF538-GbDUF538 and GaDUF538-GrDUF538, respectively. These results implied that the member expansion of *DUF538* gene family in cotton might be attributed to gene-replication event [95,96,97].

Cis-acting elements can precisely regulate the initiation and efficiency of gene transcription at specific times and in specific tissues by binding to trans-acting factors [98,99]. In the promoter regions of cotton *DUF538* genes, among the 12 types of enriched cis-acting elements, light-responsive elements are the most abundant in quantity (Figure 4). Previous studies have reported that these elements not only regulate plant growth and development via photosynthesis, photomorphogenesis, chloroplast development, and anthocyanin synthesis [100,101] but also mediate the phytohormones, thereby affecting various biological processes in plants [102]. As the most diverse hormone-responsive elements in cotton *DUF538* genes, MeJA, ABA, GA, IAA, and SA have been proven to be involved with plant growth and development [103,104], and can actively participate in the plant response process to biotic and abiotic stresses [105,106]. In addition, the enrichment of some elements related to plant defense, including defense and stress, low temperature, drought, flavonoid biosynthesis, and wound response, also provided compelling evidence for the potential functions of DUF538 proteins.

The expression profiling analysis of cotton *DUF538* genes under multiple abiotic stresses based on the published transcriptome data further verified the accuracy of the prediction of the cis-acting elements in their promoter regions. Under PEG6000 treatment from 0 h to 24 h, *GhDUF538-17*, *GhDUF538-22*, *GhDUF538-31*, *GhDUF538-34*, and *GhDUF538-55* harboring two cis-acting elements of drought responsive were induced with higher expression levels, among which only *GhDUF538-31* showed expression pattern of down-regulation first and then up-regulation, while the remaining genes presented the opposite trend of up-regulation first and then down-regulation (Figure 6d). The drought-resistant characteristics of *DUF538* genes have also been identified in the herbaceous maize and woody poplar [107,,108]. *GhDUF538-15*, *GhDUF538-22*, *GhDUF538-55*, *GhDUF538-41*, and *GhDUF538-65* were highly expressed in response to low-temperature stress (4 °C), which might be due to one or two cis-acting elements of low-temperature response (Figure 6b). Among these five *GhDUF538* genes, the former three showed first up-regulation and then down-regulation trends, while the latter two were of an opposite trend of up-regulation first, then down-regulation, and finally up-regulation. In addition to the two aforementioned abiotic stresses, nearly ten *GhDUF* genes also exhibited high expression under both high-temperature and salt stress (Figure 7a,c). Among them, *GhDUF538-17*, *GhDUF538-31*, *GhDUF538-34*, and *GhDUF538-69* all contained cis-acting element related to defense and stress response, implying their potential functions in the formation of plant abiotic stress tolerance. Additionally, some biotic stress-related elements were identified in those highly expressed *GhDUF538* genes, especially in relation to the cotton VW, of which MeJA, SA, and wound response had been reported to play significant roles in plant defense [109,110,111]. By coincidence, *GhDUF538-7* contained six MeJA and one wound-responsive elements, which was not only highly expressed in all the nine TM-1 tissues (Figure 5a), but also presented a continuously up-regulated expression pattern in response to Vd991 infection (Figure 5c). The similar tissue-specific and VW-response expression patterns were also observed in *GhDUF538-17, GhDUF538-31, GhDUF538-22*, and *GhDUF538-30*, among which the former two harbored over two MeJA elements, while the latter two harbored at least one SA element. Those above-mentioned DUF538 genes could be utilized as candidate genes for further verification research on their biological roles in cotton response to biotic and abiotic stresses, and could be used as genetic resources for improving plant stress tolerances.

It has been proven that constructing a co-expression regulatory network by analyzing the interaction relationships among proteins encoded by members of a gene family is an efficient and precise method for screening candidate genes from a large number of members of a gene family [51,112,113,114]. In the current study, protein–protein interaction analysis identified that *GhDUF538-7* and *GhDUF538-27* in Group A; *GhDUF538-31* in Group B, *GhDUF538-28* and *GhDUF538-44* in Group D; and *GhDUF538-24*, *GhDUF538-32*, and *GhDUF538-67* in Group G were hub genes (Figure 7a). However, these results were only predications based on bioinformatic and reverse-genetic analyses. When these genes were aligned with the reference genome using previous QTL studies [60,61,62,63] to correct the skewness of the above-mentioned predictions, it was observed that the hub gene *GhDUF538-7* was mapped into the physical location of the previous QTLs and was finally identified as the candidate gene. RT-PCR results indicated that *GhDUF538-7* was not an introgressive gene, since the cloned CDS and predicted AA sequences were identical between MBI9626, CCRI36, and TM-1. Via qRT-PCR experimentation on the VW-resistant MBI9626 using 0 to 15 DAI leaf samples, it was observed that *GhDUF538-7* showed the relatively complex expression pattern in response to VW infection, which was first up-regulated, then down-regulated, then up-regulated, and finally down-regulated, with its peak expression level at 3 DAI, implying its potential role in plant defense processes (Figure S1b). Having silenced the candidate gene in VW-resistant MBI9626 and WV-susceptible CCRI36, it was noted that the *TRV:DUF538-7* plants showed worse VW symptoms, significantly higher DIs, more relative pathogen biomass, more obvious vascular bundle browning, and significantly lower expression of JA-related *PR4* and *MYC2* in comparison with the TRV:00 plants (Figure 7b–e). The results indicated that *GhDUF538-7* might be a positive regulatory factor in cotton resistance against VW infection by modulating JA signaling pathways.

## 5. Conclusions

It is of great significance to investigate the gene family with unknown functions; therefore, genome-wide analyses of the *DUF538* gene family were conducted on four representative *Gossypium* species, resulting in the identification of 214 members that were classified into seven groups. Through the comparative analyses of exon–intron structures and conserved motifs, conservatism and diversity were simultaneously observed between the different groups. More gene-replication pairs were identified in both intraspecific and interspecific alignments, and these collinearity analyses provided the supporting evidence for the inference that gene-replication events might be the driving force for the expansion of the cotton *DUF538* gene family. Plenty of cis-acting elements responding to biotic and abiotic stresses were identified in their promoter regions, whose accuracy of functional prediction was further verified by expression-pattern analyses on the response of cotton *DUF538* genes to low-temperature, high-temperature, salt, and drought stresses, together with VW infection. As one of the hub genes in the protein–protein interaction network, *GhDUF538-7* was also identified to be located in overlapping intervals of multiple published QTLs for VW resistance and was therefore selected to perform VIGS experimentation. In both the VW-resistant MBI9626 and VW-susceptible CCRI36, silencing *DUF538-7* resulted in more severe VW symptoms, significantly higher DIs, more accumulated fungal content, and more severe vascular bundle browning, indicating its potentially positive role in plant defense. Additionally, by comparing the expression data of *TRV:DUF538-7* and *TRV:00* plants, it was found that the relative expression levels of JA-related *PR4* and *MYC2* genes were also significantly down-regulated along with the silencing of the candidate gene, implying a potential synergistic regulatory relationship between them. This study not only provided abundant evidence for elucidating the molecular mechanism of *GhDUF538-7* in response to VW infection, but it also laid a solid foundation for synergistic improvement of cotton yield, fiber quality, and stress resistance in the future.

## Data Availability

The original contributions presented in the study are included in the article/Supplementary Materials; further inquiries can be directed to the corresponding authors.

## Supplementary Materials

The following supporting data can be downloaded at: https://www.mdpi.com/article/10.3390/plants15142148/s1, Figure S1: The sequence cloning and alignment, responding pattern to VW infection of *DUF538*, and albinism phenotype of *GhCLA1*. (a) CDS alignment of *DUF538* between MBI9626, Hai1, Hai7124, CCRI36, and TM-1. (b) The alignment of protein sequences of DUF538 between MBI9626, Hai1, Hai7124, CCRI36, and TM-1. (c) The gel electrophoresis image of CDS cloning of *DUF538* in MBI9626, Hai1, and CCRI36. (d) The expression pattern of DUF538 at different time points in response to VW infection. (e) The albinism phenotype of *GhCLA1.* Figure S2: The relative expression-level detection of JA-related *PR4* and *MYC2* in *TRV:DUF538-7* and *TRV:00* plants. Table S1: Characterization of cotton DUF538 proteins.
