# Supplementary material for: Domains of Unknown Function 538-7 Regulates Cotton Resistance to Verticillium Wilt by Mediating Jasmonate Signaling Pathways
Source: Plants (Basel). 2026 Jul 12;15(14):2148. doi: 10.3390/plants15142148 (PMC13415095; doi:10.3390/plants15142148)
Supplement: Supplementary file 1 [file plants-15-02148-s001.zip › Table S1.pdf]

Table S1 Characterization of cotton DUF538 proteins

Gene ID	Len (aa)	MW (KDa)	pI	Ins	AI	GRAVY	Sub.	Gene ID	Len (aa)	MW (KDa)	pI	Ins	AI	GRAVY	Sub.
GaDUF538-1	149	16.62	5.44	34.51	108.52	0.02	cyto	GrDUF538-1	184	20.62	4.94	31.85	90.6	0.01	extr
GaDUF538-2	244	27.25	9.25	34.6	83.52	-0.39	cyto	GrDUF538-2	164	18.16	6.41	38.13	113.41	0.2	chlo
GaDUF538-3	166	19.02	9.08	50.21	78.61	-0.33	nucl	GrDUF538-3	170	19	7.73	28.55	92.82	-0.32	cyto
GaDUF538-4	146	16.5	8.45	24.66	88.15	-0.23	cyto	GrDUF538-4	167	18.92	9.57	37.25	78.08	-0.44	chlo
GaDUF538-5	107	12.46	9.33	21.51	86.54	-0.26	cyto	GrDUF538-5	174	19.02	5.14	40.71	99.14	0.16	plas
GaDUF538-6	158	17.4	8.89	27	87.03	-0.14	chlo	GrDUF538-6	158	17.04	4.2	38.04	97.53	0.28	extr
GaDUF538-7	136	15.07	9.05	26.75	95.96	-0.04	cyto	GrDUF538-7	190	21.45	5.12	36.2	105.58	0.13	extr
GaDUF538-8	210	23.71	8.26	30.29	95.05	0.19	chlo	GrDUF538-8	182	20.66	4.7	43.34	103.3	0.07	extr
GaDUF538-9	169	18.91	7.76	42.24	115.27	0.12	chlo	GrDUF538-9	169	18.8	5.84	39.34	89.88	-0.28	cyto
GaDUF538-10	162	17.79	6.05	52.89	109.38	0.17	extr	GrDUF538-10	170	19.18	5.04	30.3	84.82	0.16	cyto
GaDUF538-11	146	16.86	8.39	48.99	88.77	-0.33	cyto	GrDUF538-11	166	18.79	9.08	55.12	74.52	-0.32	chlo
GaDUF538-12	147	16.92	5.48	50.41	68.37	-0.6	nucl	GrDUF538-12	137	15.35	7.84	26.98	98.83	-0.08	cyto
GaDUF538-13	136	15.16	8.71	32.14	97.43	-0.1	chlo	GrDUF538-13	170	19.06	8.73	22.86	85.35	-0.32	nucl
GaDUF538-14	197	21.13	4.68	33.02	99.95	0.25	extr	GrDUF538-14	178	19.82	7.57	27.87	84.78	0.08	chlo
GaDUF538-15	184	20.63	4.94	32.52	87.93	-0.02	extr	GrDUF538-15	183	20.44	7.71	46.3	108.58	0.19	chlo
GaDUF538-16	175	19.1	5.65	35.66	100.29	0.18	plas	GrDUF538-16	159	18.26	5.82	45.51	59.43	-0.7	cyto
GaDUF538-17	158	17.23	4.43	36.3	96.27	0.22	extr	GrDUF538-17	155	16.88	4.99	26.89	94.39	0.23	extr
GaDUF538-18	182	20.7	4.64	41.34	103.3	0.04	extr	GrDUF538-18	184	20.78	4.84	38.02	97.99	0.11	extr
GaDUF538-19	169	18.81	6.2	40.61	90.47	-0.26	extr	GrDUF538-19	169	18.37	4.64	27.53	96.86	0.1	extr
GaDUF538-20	170	19.26	5.28	32.1	84.82	0.08	nucl	GrDUF538-20	168	18.47	5.05	31.03	99.17	0.19	extr
GaDUF538-21	137	15.34	8.65	25.26	98.83	-0.12	cyto	GrDUF538-21	210	23.65	8.28	32.67	99.24	0.24	chlo
GaDUF538-22	170	19.06	8.73	22.86	85.35	-0.32	nucl	GrDUF538-22	158	17.57	9.38	31.82	92.47	-0.16	cyto
GaDUF538-23	108	12.5	9.23	45.49	99.17	-0.1	nucl	GrDUF538-23	169	18.95	7.76	42.63	110.65	0.08	chlo

Gene ID	Len (aa)	MW (KDa)	pI	Ins	AI	GRAVY	Sub.	Gene ID	Len (aa)	MW (KDa)	pI	Ins	AI	GRAVY	Sub.
GaDUF538-24	155	17.34	5.01	56.16	99.94	0.07	chlo	GrDUF538-24	158	17.32	8.53	26.05	86.39	-0.12	chlo
GaDUF538-25	183	20.44	7.79	44.28	112.84	0.21	chlo	GrDUF538-25	162	17.79	6.05	52.89	109.38	0.16	extr
GaDUF538-26	178	19.74	7.54	28.77	84.78	0.13	chlo	GrDUF538-26	147	16.9	5.54	47.17	68.37	-0.6	nucl
GaDUF538-27	184	20.85	4.85	36.51	95.87	0.05	extr	GrDUF538-27	136	15.19	8.71	31.04	98.82	-0.09	cyto
GaDUF538-28	141	15.29	5.26	41.27	87.87	0.05	extr	GrDUF538-28	141	15.94	9.24	32.23	86.31	-0.3	chlo
GaDUF538-29	159	18.19	5.82	41.75	56.98	-0.71	nucl	GrDUF538-29	155	17.34	5.17	55.35	100.58	0.08	chlo
GaDUF538-30	168	18.43	4.75	28.41	102.08	0.25	extr	GrDUF538-30	146	16.48	8.45	23.61	88.15	-0.22	cyto
GaDUF538-31	169	18.32	4.52	26.64	96.27	0.11	extr	GrDUF538-31	107	12.5	9.14	24.96	80.09	-0.33	cyto
GaDUF538-32	178	20.03	5.05	30.71	99.55	0.1	chlo	GrDUF538-32	178	20.15	9.5	32.06	84.21	-0.31	chlo
GaDUF538-33	178	20.12	9.5	32.49	84.21	-0.32	chlo	GrDUF538-33	173	19.35	5.36	43.13	101.39	0.13	extr
GaDUF538-34	150	16.77	6.75	48.22	87.67	-0.37	nucl	GrDUF538-34	150	16.79	7.67	46.4	87.67	-0.38	chlo
GaDUF538-35	173	19.36	5.2	43.97	103.64	0.16	vacu	GrDUF538-35	178	19.94	5.5	27.79	98.99	0.13	extr
GaDUF538-36	166	18.53	6.2	35.75	89.7	-0.32	cyto	GrDUF538-36	166	18.51	6.2	37.17	89.7	-0.32	cyto
GaDUF538-37	170	18.82	6.83	26.4	92.82	-0.24	cyto	GrDUF538-37	197	21.14	4.76	33.62	102.39	0.3	extr
GbDUF538-1	164	18.13	6.42	38.41	111.04	0.15	chlo	GhDUF538-1	164	18.13	6.42	38.41	111.04	0.15	chlo
GbDUF538-2	187	20.76	6.95	25.94	97.38	-0.11	cyto	GhDUF538-2	170	18.82	6.83	26.4	92.82	-0.24	cyto
GbDUF538-3	166	19.02	9.08	50.21	78.61	-0.33	nucl	GhDUF538-3	166	19.02	9.08	50.21	78.61	-0.33	nucl
GbDUF538-4	228	25.73	9.76	35.04	84.17	-0.34	chlo	GhDUF538-4	228	25.75	9.76	34.2	82.46	-0.35	chlo
GbDUF538-5	169	18.91	7.76	42.24	115.27	0.12	chlo	GhDUF538-5	164	18.32	7.77	44.46	117.56	0.16	chlo
GbDUF538-6	146	16.51	8.45	25.82	88.84	-0.23	cyto	GhDUF538-6	146	16.53	8.45	28.97	88.84	-0.23	cyto
GbDUF538-7	158	17.41	8.89	27.54	87.66	-0.14	chlo	GhDUF538-7	158	17.4	8.89	27	87.03	-0.14	chlo
GbDUF538-8	136	15.08	9.05	26.75	95.96	-0.05	cyto	GhDUF538-8	136	15.08	9.05	26.75	95.96	-0.05	cyto
GbDUF538-9	162	17.79	6.05	53.42	110	0.17	extr	GhDUF538-9	195	22.01	6.04	30.01	97.33	0.25	chlo
GbDUF538-10	146	16.78	8.73	46.24	88.77	-0.3	cyto	GhDUF538-10	162	17.79	6.05	53.42	110	0.17	extr

Gene ID	Len (aa)	MW (KDa)	pI	Ins	AI	GRAVY	Sub.	Gene ID	Len (aa)	MW (KDa)	pI	Ins	AI	GRAVY	Sub.
GbDUF538-11	149	17.18	5.77	52.05	67.45	-0.6	nucl	GhDUF538-11	146	16.84	8.73	46.35	88.77	-0.33	cyto
GbDUF538-12	136	15.16	8.71	32.14	97.43	-0.1	chlo	GhDUF538-12	149	17.18	5.77	52.05	67.45	-0.6	nucl
GbDUF538-13	197	21.07	4.76	33.37	101.42	0.29	extr	GhDUF538-13	136	15.16	7.9	32.46	97.43	-0.11	chlo
GbDUF538-14	184	20.64	4.86	32.52	88.48	-0.05	extr	GhDUF538-14	197	21.07	4.76	33.37	101.42	0.29	extr
GbDUF538-15	177	19.33	5.65	35.37	103.56	0.22	extr	GhDUF538-15	184	20.59	4.94	32.52	89.51	-0.01	extr
GbDUF538-16	158	17.23	4.43	36.3	96.27	0.22	extr	GhDUF538-16	175	19.1	5.65	35.66	100.29	0.18	plas
GbDUF538-17	182	20.63	4.7	41.34	103.3	0.06	extr	GhDUF538-17	158	17.23	4.43	36.3	96.27	0.22	extr
GbDUF538-18	172	19.06	6.75	35.05	94.01	-0.13	cyto	GhDUF538-18	205	23.58	5.45	35.95	97.41	-0.05	extr
GbDUF538-19	170	19.26	5.28	32.1	84.82	0.08	nucl	GhDUF538-19	172	19.06	6.75	35.05	94.01	-0.13	cyto
GbDUF538-20	137	15.32	8.65	22.46	100.95	-0.11	cyto	GhDUF538-20	170	19.26	5.28	32.1	84.82	0.08	nucl
GbDUF538-21	170	19.06	8.73	22.86	85.35	-0.32	nucl	GhDUF538-21	137	15.32	8.65	22.46	100.95	-0.11	cyto
GbDUF538-22	138	15.47	4.8	53.51	85.43	-0.24	chlo	GhDUF538-22	170	19.06	8.73	22.86	85.35	-0.32	nucl
GbDUF538-23	141	16.21	9.52	43.63	90.5	-0.38	nucl	GhDUF538-23	155	17.37	4.91	56.71	98.06	0.02	chlo
GbDUF538-24	159	18.2	5.82	42	53.9	-0.76	nucl	GhDUF538-24	159	18.17	5.82	40.87	51.45	-0.76	cyto
GbDUF538-25	138	14.96	6.55	37.32	84.13	0.12	extr	GhDUF538-25	138	15.02	6.55	35.38	86.96	0.16	extr
GbDUF538-26	184	20.78	4.93	36.05	95.87	0.06	extr	GhDUF538-26	184	20.85	4.85	36.51	95.87	0.05	extr
GbDUF538-27	178	19.78	6.57	29.49	84.21	0.11	chlo	GhDUF538-27	178	19.79	6.57	30.09	84.78	0.11	chlo
GbDUF538-28	184	20.51	7.79	43.05	112.77	0.22	chlo	GhDUF538-28	184	20.51	7.79	43.05	112.77	0.22	chlo
GbDUF538-29	142	15.52	4.44	29.63	103.59	0.24	vacu	GhDUF538-29	175	19.03	4.65	29.56	109.66	0.26	plas
GbDUF538-30	178	20.03	5.05	30.71	99.55	0.1	chlo	GhDUF538-30	178	20.03	5.05	30.71	99.55	0.1	chlo
GbDUF538-31	178	20.12	9.5	32.49	84.21	-0.32	chlo	GhDUF538-31	178	20.12	9.5	32.49	84.21	-0.32	chlo
GbDUF538-32	150	16.77	6.75	48.22	87.67	-0.37	nucl	GhDUF538-32	150	16.77	6.75	48.22	87.67	-0.37	nucl
GbDUF538-33	159	17.92	4.84	41.65	99.87	0.16	vacu	GhDUF538-33	173	19.36	5.07	43.97	103.64	0.16	extr
GbDUF538-34	166	18.53	6.2	35.75	89.7	-0.32	cyto	GhDUF538-34	166	18.53	6.2	35.75	89.7	-0.32	cyto

Gene ID	Len (aa)	MW (KDa)	pI	Ins	AI	GRAVY	Sub.	Gene ID	Len (aa)	MW (KDa)	pI	Ins	AI	GRAVY	Sub.
GbDUF538-35	164	18.17	6.9	38.59	111.04	0.14	chlo	GhDUF538-35	164	18.17	6.9	38.59	111.04	0.14	chlo
GbDUF538-36	170	19	7.73	28.55	92.82	-0.32	cyto	GhDUF538-36	170	19.03	7.73	28.39	93.94	-0.31	cyto
GbDUF538-37	166	18.82	9.08	55.23	72.17	-0.33	chlo	GhDUF538-37	166	18.84	8.91	55	73.92	-0.36	chlo
GbDUF538-38	167	18.78	9.65	34.17	78.08	-0.42	chlo	GhDUF538-38	148	16.56	9.8	30.16	78.24	-0.31	nucl
GbDUF538-39	146	16.48	8.45	23.61	88.15	-0.22	cyto	GhDUF538-39	146	16.47	8.45	23.61	88.15	-0.22	cyto
GbDUF538-40	146	17.01	9.13	25.34	74.73	-0.5	cyto	GhDUF538-40	146	17.05	9.13	26.37	78.7	-0.44	cyto
GbDUF538-41	158	17.37	8.89	25.51	85.76	-0.15	chlo	GhDUF538-41	158	17.37	8.89	25.51	85.76	-0.15	chlo
GbDUF538-42	136	15.17	7.9	25.59	95.96	-0.08	cyto	GhDUF538-42	136	15.12	8.73	24.87	95.96	-0.04	cyto
GbDUF538-43	167	18.48	6.4	27.07	97.96	0.23	chlo	GhDUF538-43	210	23.64	8.27	30.38	99.67	0.25	chlo
GbDUF538-44	169	18.92	7.76	41.9	111.24	0.09	chlo	GhDUF538-44	169	18.92	7.76	41.9	111.24	0.09	chlo
GbDUF538-45	162	17.79	6.05	52.89	109.38	0.16	extr	GhDUF538-45	161	17.73	6.05	52.69	110.06	0.17	extr
GbDUF538-46	146	16.66	6.84	46.9	88.08	-0.3	cyto	GhDUF538-46	146	16.66	6.84	46.9	88.08	-0.3	cyto
GbDUF538-47	147	16.87	5.28	45.28	68.37	-0.6	nucl	GhDUF538-47	147	16.9	5.54	47.17	68.37	-0.6	nucl
GbDUF538-48	136	15.19	8.71	31.04	98.82	-0.09	cyto	GhDUF538-48	136	15.19	8.71	31.04	98.82	-0.09	cyto
GbDUF538-49	164	17.65	5.62	36	97.44	0.26	chlo	GhDUF538-49	184	20.66	4.94	32.89	88.48	0	extr
GbDUF538-50	184	20.61	4.83	32.89	90.6	0.05	extr	GhDUF538-50	158	17.07	4.2	35.26	96.9	0.26	extr
GbDUF538-51	174	19.02	4.96	41.25	99.14	0.16	plas	GhDUF538-51	182	20.67	4.8	39.6	102.75	0.06	extr
GbDUF538-52	158	17.04	4.31	33.68	96.9	0.25	extr	GhDUF538-52	169	18.78	5.84	39.68	89.29	-0.27	cyto
GbDUF538-53	182	20.68	4.8	42.6	103.3	0.07	extr	GhDUF538-53	170	19.2	5.25	31.43	84.82	0.12	cyto
GbDUF538-54	169	18.78	5.84	39.68	89.29	-0.27	cyto	GhDUF538-54	137	15.31	7.84	25.02	96.72	-0.12	cyto
GbDUF538-55	170	19.2	5.25	31.43	84.82	0.12	cyto	GhDUF538-55	170	19.06	8.73	22.86	85.35	-0.32	nucl
GbDUF538-56	137	15.35	7.84	26.98	98.83	-0.08	cyto	GhDUF538-56	144	16.07	5.14	63.77	108.26	0.19	chlo
GbDUF538-57	170	19.06	8.73	22.86	85.35	-0.32	nucl	GhDUF538-57	158	18.29	9.35	35.71	83.23	-0.45	nucl
GbDUF538-58	144	16.07	5.14	63.77	108.26	0.19	chlo	GhDUF538-58	145	16.75	6.33	44.84	53.79	-0.75	cyto

Gene ID	Len (aa)	MW (KDa)	pI	Ins	AI	GRAVY	Sub.	Gene ID	Len (aa)	MW (KDa)	pI	Ins	AI	GRAVY	Sub.
GbDUF538-59	158	18.29	9.35	35.71	83.23	-0.45	nucl	GhDUF538-59	155	16.85	4.99	28.13	91.87	0.2	extr
GbDUF538-60	159	18.3	5.99	48.72	61.89	-0.66	cyto	GhDUF538-60	209	23.43	5.51	43.39	99.33	0.08	extr
GbDUF538-61	155	16.88	4.99	26.89	94.39	0.23	extr	GhDUF538-61	178	19.72	7.57	26.9	84.78	0.11	chlo
GbDUF538-62	184	20.78	4.84	38.02	97.99	0.11	extr	GhDUF538-62	183	20.44	7.71	46.3	108.58	0.19	chlo
GbDUF538-63	178	19.82	8.25	27.85	84.78	0.09	chlo	GhDUF538-63	168	18.2	4.59	27.27	95.12	0.1	extr
GbDUF538-64	183	20.46	6.82	45.95	106.99	0.15	chlo	GhDUF538-64	193	21.32	4.97	29.65	99.95	0.14	extr
GbDUF538-65	164	18	4.55	29.13	101.59	0.13	plas	GhDUF538-65	178	19.97	5.5	28.17	98.99	0.12	extr
GbDUF538-66	180	20.32	6.81	25.38	101.11	0.18	chlo	GhDUF538-66	178	20.02	9.5	30.59	82.58	-0.32	chlo
GbDUF538-67	178	20.09	9.43	34.32	84.21	-0.32	chlo	GhDUF538-67	150	16.81	7.67	49.86	85.07	-0.43	extr
GbDUF538-68	150	16.79	7.67	46.4	87.67	-0.38	chlo	GhDUF538-68	173	19.34	5.2	43.97	101.39	0.13	vacu
GbDUF538-69	173	19.34	5.2	43.97	101.39	0.13	vacu	GhDUF538-69	138	15.48	8.46	38.24	93.84	-0.14	cyto
GbDUF538-70	166	18.51	6.2	37.17	89.7	-0.32	cyto	GhDUF538-70	89	10.28	9.41	31.34	83.26	-0.41	nucl
