# Supplementary figures and images for: Domains of Unknown Function 538-7 Regulates Cotton Resistance to Verticillium Wilt by Mediating Jasmonate Signaling Pathways
Source: Plants (Basel). 2026 Jul 12;15(14):2148. doi: 10.3390/plants15142148 (PMC13415095; doi:10.3390/plants15142148)

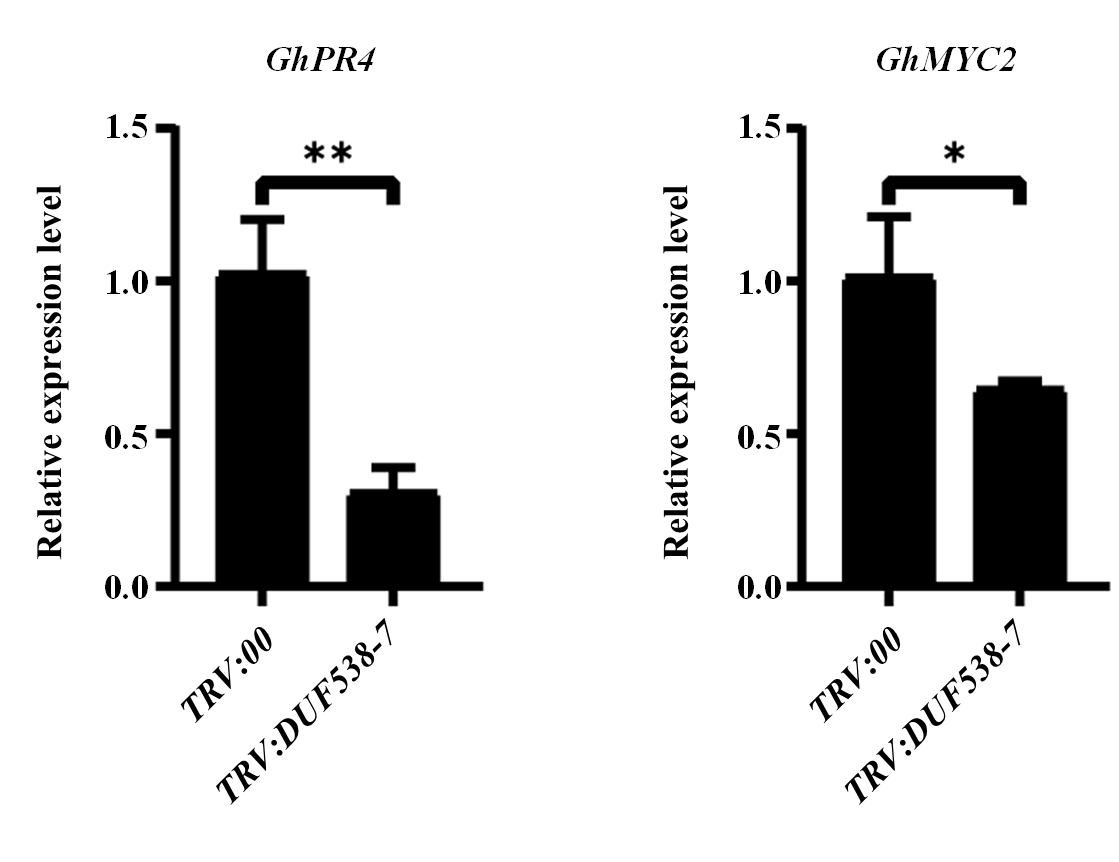

Supplement: Supplementary file 1 [file plants-15-02148-s001.zip › Figure S2.tif]
